# Lapatinib in combination with capecitabine versus continued use of trastuzumab in breast cancer patients with trastuzumab-resistance: a retrospective study of a Chinese population

**DOI:** 10.1186/s12885-020-6639-4

**Published:** 2020-03-29

**Authors:** Fan Yang, Xiang Huang, Chunxiao Sun, Jianbin Li, Biyun Wang, Min Yan, Feng Jin, Haibo Wang, Jin Zhang, Peifen Fu, Tianyu Zeng, Jian Wang, Wei Li, Yongfei Li, Mengzhu Yang, Jun Li, Hao Wu, Ziyi Fu, Yongmei Yin, Zefei Jiang

**Affiliations:** 1 Department of Oncology, The First Affiliated Hospital of Nanjing Medical University, 300 Guangzhou Road, Nanjing, 210029 People’s Republic of China; 2 The First Clinical College of Nanjing Medical University, Nanjing, 210029 People’s Republic of China; 3 Department of Breast Cancer, The 307 Hospital of Chinese People’s Liberation Army, Beijing, 100000 People’s Republic of China; 4 Department of Medical Oncology, Fudan University Shanghai Cancer Center, Shanghai, 200032 People’s Republic of China; 5 Department of Breast Cancer, Henan Cancer Hospital, Zhengzhou, People’s Republic of China; 6 Department of Breast Surgery, the First Affiliated Hospital of China Medical University, Shenyang, People’s Republic of China; 7 Department of Breast Cancer Center, Affiliated Hospital of Medical College Qingdao University, Qingdao, People’s Republic of China; 8 Department of Breast Cancer, Tianjin Medical University Cancer Institute and Hospital, Tianjin, People’s Republic of China; 9 Department of Breast Center, The First Affiliated Hospital, College of Medicine, Zhejiang University, Hangzhou, People’s Republic of China; 10 Department of Oncology, The Third Affiliated Hospital of Soochow University, Changzhou, People’s Republic of China; 11 Nanjing Maternal and Child Health Medical Institute, Obstetrics and Gynecology Hospital Affiliated to Nanjing Medical University, Nanjing, 210004 People’s Republic of China; 12 Jiangsu Key Lab of Cancer Biomarkers, Prevention and Treatment, Collaborative Innovation Center for Cancer Medicine, Nanjing Medical University, Nanjing, 211166 People’s Republic of China

**Keywords:** Lapatinib, Trastuzumab, Resistance, Breast cancer

## Abstract

**Background:**

The efficacy and safety of lapatinib plus capecitabine (LC or LX) versus trastuzumab plus chemotherapy in patients with HER-positive metastatic breast cancer who are resistant to trastuzumab is unknown.

**Methods:**

We retrospectively analyzed data from breast cancer patients who began treatment with regimens of lapatinib plus capecitabine (LC or LX) or trastuzumab beyond progression (TBP) at eight hospitals between May 2010 and October 2017.

**Results:**

Among 554 patients who had developed resistance to trastuzumab, the median PFS (progression free survival) was 6.77 months in the LX group compared with 5.6 months in the TBP group (hazard ratio 0.804; 95% CI, 0.67 to 0.96; *P* = 0.019). The central nervous system progression rate during treatment was 5.9% in the LX group and 12.5% in the TBP group (*P* = 0.018).

**Conclusion:**

The combination of lapatinib and capecitabine showed a prolonged PFS relative to TBP in patients who had progressed on trastuzumab.

## Background

Breast cancer is one of the most common invasive cancers and is expected to account for 14% of all cancer deaths in women worldwide [1]. Activation and overexpression of epidermal growth factor receptor (EGFR, also known as ErbB) family members, including EGFR (ErbB1 or HER1), HER3 (ErbB3), HER4 (ErbB4), and HER2 (ErbB2), govern multiple important cellular processes in breast cancer. Activation of HER2, a tyrosine kinase receptor, induces homo- and heterodimerization, which leads to the activation of downstream effectors and pathways such as PI3K/AKT and RAS/MAP K[2].

Amplification of the HER2 gene and/or overexpression of its protein product occurs in approximately 20–25% of breast cancer s[3]. Clinically, HER2-positive tumors are characterized by an aggressive clinical course and a poor overall prognosi s[4]. The introduction of the anti-HER2 monoclonal antibody trastuzumab into clinical practice has dramatically improved the poor prognosis of this population of patient s[5–7]. Trastuzumab binds to the extracellular domain of the HER2 receptor and prevents receptor homo- and heterodimerization, thereby inhibiting the activation of downstream oncogenic signalin g[8]. Adding trastuzumab to the treatment regimen is the standard approach for treating HER-2 positive metastatic breast cancer. However, despite its overall clinical efficacy, de novo and acquired resistance to trastuzumab administration have been observe d[9]. The development of distant metastases to liver, bone, lung and brain has become a major challenge in the management of patients with HER-2 positive breast cancer, probably due to their longer life expectancy and acquired trastuzumab resistanc e[10]. Therefore, there is an urgent need to develop a new strategy for salvage therapy of patients who have developed resistance to trastuzumab.

However, consensus guidelines on targeted treatment for resistance in HER2-positive breast cancer are not availabl e[11, 12]. Combinations of anti-HER2 agents with chemotherapy, anti-HER2/HER3 dimerization agents, or inhibitors of its downstream signaling pathways might improve patient prognosi s[13]. Fujimoto-Ouchi demonstrated that trastuzumab in combination with taxanes or capecitabine showed antitumor activity in a trastuzumab-resistant mode l[14].

The GBG 26/BIG 3–05 enrolled patients with HER2-positive metastatic breast cancer (stage IV) that progressed during treatment with trastuzumab. Among these patients, 78 patients were randomly assigned to receive capecitabine, and 78 patients were assigned to capecitabine plus trastuzumab. The results showed that the median TTPs were 5.6 months vs 8.2 months, *P* = 0.033 8[15]. In a similar study, patients who received trastuzumab treatment beyond progression (TBP) had a longer median OS than those who terminated trastuzumab (21.3 months vs 4.6 months (*P*<0.0001 )[16]. Taken together, the findings of these studies suggest that a clinical benefit has been observed for treatment with trastuzumab beyond progression.

Lapatinib, an orally active small-molecule tyrosine kinase inhibitor, has shown non-cross-resistance with trastuzumab. It binds reversibly to the cytoplasmic domains of both EGFR and HER2, which then blocks the activating signaling cascades in the MAPK and PI3K pathway s[17]. Given its unique mechanistic function, lapatinib might be a suitable treatment option for HER2-positive MBCs that have become resistant to suppression by trastuzumab.

Some studies have also shown that the phosphorylation of p95 HER2 (a truncated version lacking the extracellular domain) and the formation of heterodimers between HER2 and other members of the HER family might be inhibited by lapatinib but not trastuzuma b[18, 19]. In the EGF100151 trial, lapatinib plus capecitabine reduced the hazard for time-to-disease progression (hazard ratio 0.49; 95% CI 0.34–0.71; *P* < 0.001) in cases of HER2-positive breast cancer that progressed on anthracycline, a taxane and trastuzuma b[11, 20].

In 2010, the US FDA approved the use of lapatinib in combination with capecitabine for the treatment of patients with HER2-positive MBC. In addition, lapatinib in combination with capecitabine shows excellent activity against central nervous system (CNS) metastases. The results of one study suggested that patients with brain metastases achieved significantly longer overall survival in the lapatinib group compared with those on the trastuzumab-based therapy (19.1 vs 12 months, *P* = 0.039 )[21].

Clinical trials have demonstrated that other HER-2 targeted agents, such as T-DM1 and pertuzumab, have shown efficacy in patients pretreated with trastuzuma b[22, 23]. However, these regimens remain unavailable in China. Therefore, trastuzumab plus chemotherapy or switching to the lapatinib plus capecitabine regimen are common options for Chinese patients who have developed resistance to trastuzumab. No compelling evidence indicates if certain patients benefit more from the continuation of trastuzumab compared with switching to lapatinib. In the present analysis, we compare the clinical outcome of continuing trastuzumab treatment or replacing trastuzumab with lapatinib for metastatic breast cancer (MBC) patients who are resistant to trastuzumab.

## Methods

### Patients

We retrospectively reviewed the medical records of HER2-positive metastatic breast cancer patients at CSCO breast cancer database (research number: CSCO BC RWS1801) from May 2010 to October 2017. HER-2 status was considered positive if an immunohistochemistry (IHC) test showed +++ or if HER2 gene amplification was found by fluorescence in situ hybridization. Female patients who received lapatinib plus capecitabine or trastuzumab plus chemotherapy after developing resistance to trastuzumab were included. Primary resistance was defined as new recurrences diagnosed during or within 12 months after the end of (neo) adjuvant trastuzumab or progression was observed at the first radiological reassessment at 8–12 weeks or within 3 months of initiating trastuzumab therapy for metastatic disease. Secondary resistance was defined as disease progression of metastatic cancer occurring while on trastuzumab-containing regimens that initially achieved a disease response or stabilization at the first radiological assessment. We excluded patients whose therapeutic regimen had been administered beyond the third line for recurrent metastatic breast cancer and those that received anti-HER2 therapies other than trastuzumab. Patients with central nervous system metastases had to have previously been treated with radiotherapy or surgery. All patients who had at least one measurable disease lesion and a tumor response were evaluated according to the Response Evaluation Criteria in Solid Tumors 1.1.

### Endpoint

The primary endpoint was PFS, defined as the time from the initiation of TBP or LX until the earliest date of disease progression or death. Secondary outcomes included ORR (the ratio of patients who had complete or partial tumor remission) and CBR (clinical benefit rate), defined as the ratio of patients who had complete or partial tumor remission or stable disease for more than 6 months.

### Statistical analysis

Statistical analyses were performed using SPSS version 24.0 (SPSS Inc., Chicago, IL, USA). A two-tailed *P* < 0.05 was defined as significant. Kaplan-Meier estimates were used to compare PFS using the log-rank test. Comparisons of ORR, CBR, and central nervous system progression rates were conducted using chi-square tests. Categorical variables were compared between the groups by chi-square tests. The effects of various baseline covariates on PFS were analyzed by Cox regression modeling.

## Results

### Patient characteristics

A total of 554 patients were identified and the median follow-up time was 15 months. The demographic characteristics of the two groups are shown in Table 1, and most variables were well-balanced. A higher proportion of patients in the TBP group were older than 50 years and had HR-positive tumors. A total of 94 (36.9%) patients received lapatinib plus capecitabine (LX), and 164 (54.8%) patients received trastuzumab beyond progression (TBP) as second-line treatment (*P* < 0.001). While on third-line treatment, 124 (48.6%) patients received lapatinib plus capecitabine (LX) and 92 (30.8%) patients received trastuzumab beyond progression (TBP) (*P* = 0.001), which indicated more patients received LX in later lines. The predominant chemotherapy combined with trastuzumab was taxane (Table 2).

**Table 1:** Baseline characteristics

Parameter	LX	TBP	*P* value
	(*N* = 255)	(*N* = 299)	
Age (year)			
< 50	137(53.7%)	161(53.8%)	0.977
≥ 50	59(23.1%)	95(31.8%)	0.024
Unknown	59(23.1%)	43(14.4%)	0.008
Menopausal status			
Premenopausal	40(15.7%)	68(20.7%)	0.126
Postmenopausal	182(71.4%)	204(68.2%)	0.422
Unknown	33(12.9%)	27(9%)	0.14
HR Status			
Negative	136(53.3%)	145(48.5%)	0.256
Positive	92(36.1%)	139(46.5%)	0.013
Unknown	27(10.6%)	15(5%)	0.014
Stage IV at initial diagnosis	32(12.5%)	55(18.4%)	0.059
Number of metastatic sites			
< 3	178(69.8%)	190(63.5%)	0.12
≥ 3	77(30.2%)	109(36.5%)	
Metastases			
Lung	123(48.2%)	162(54.2%)	0.163
Liver	109(42.7%)	143(47.8%)	0.213
Bone	62(24.3%)	86(28.8%)	0.238
Brain	24(9.4%)	34(11.4%)	0.453
Other	131(51.4%)	150(50.2%)	0.78
Resistance			
Primary	96(37.6%)	109(38.1%)	0.772
Secondary	159(62.4%)	190(61.9%)	
Treatment line			
1	37(14.5%)	43(14.4%)	0.966
2	94(36.9%)	164(54.8%)	<0.001
3	124(48.6%)	92(30.8%)	0.001
Previous therapy			
Hormonal			
Adjuvant	76(29.8%)	96(32.1%)	0.559
Metastatic	60(23.5%)	91(30.4%)	0.069
Radiotherapy			
Adjuvant	86(33.7%)	104(34.8%)	0.794
Metastatic	44(17.3%)	54(18.1%)	0.804
Previous trastuzumab failure			
Adjuvant	37(14.5%)	43(14.4%)	0.966
Metastatic	218(85.5%)	256(85.6%)	
Previous trastuzumab treatment			
Adjuvant	78(30.6%)	67(22.4%)	0.029
Advanced disease only	177(69.4%)	232(77.6%)

**Table 2:** chemotherapy combined with trastuzumab

	Patients (*N* = 299)
Taxane	146(48.8%)
Vinorelbine	33(11%)
Gemcitabine	75(25.1%)
Cisplatin	60(20.1%)
Pemetrexed	8(2.7%)
Carboplatin	6(2%)
Capecitabine	71(23.7%)

### Efficacy

**Fig. 1:**
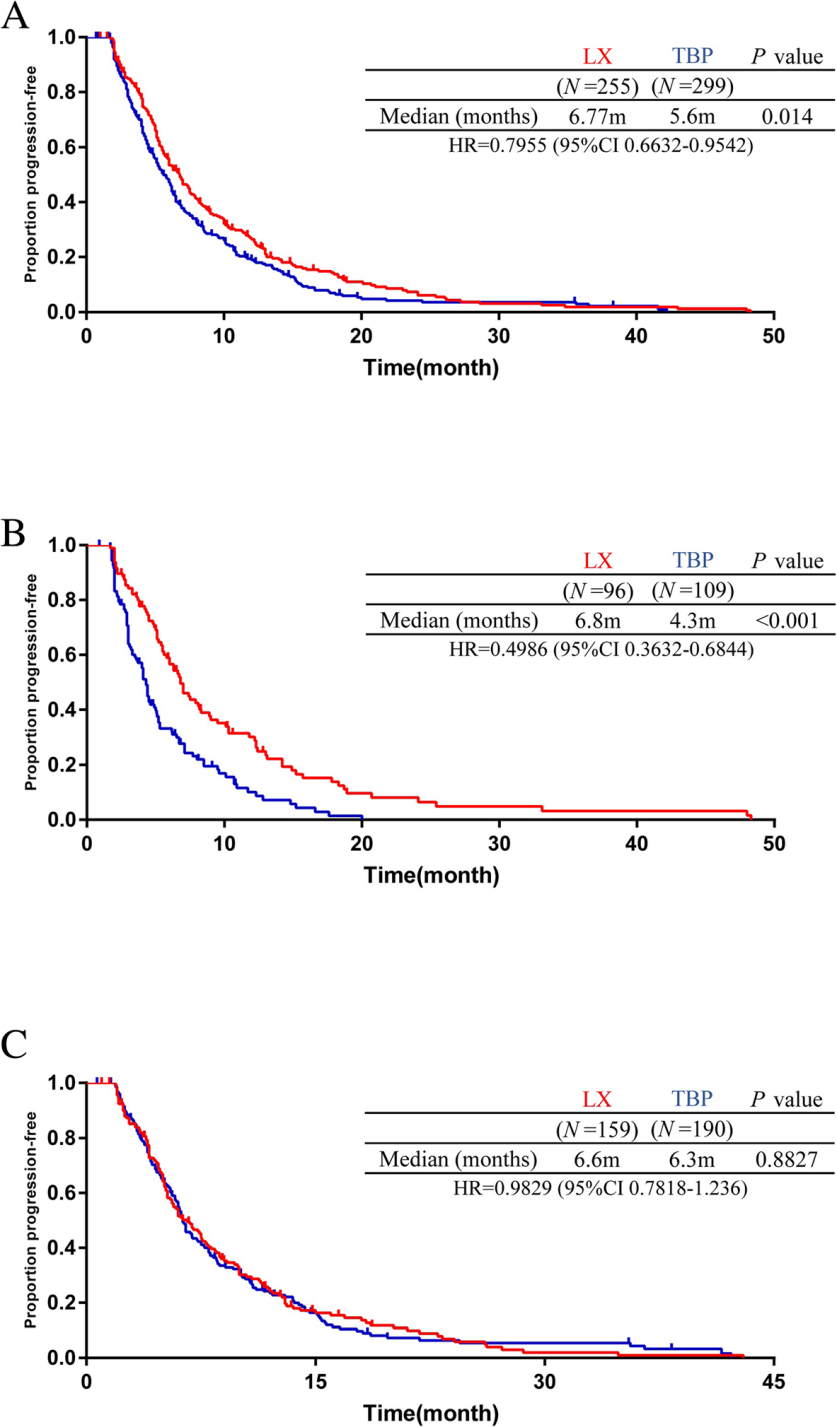
Kaplan-Meier analysis of progression-free survival (**a**) PFS in all patients (**b**) PFS in the primary resistant population (**c**) PFS in the secondary resistant population. CI, confidence interval; HR, hazard ratio; m, months; PFS, progression-free survival; LX, lapatinib plus capecitabine; TBP, trastuzumab beyond progression

The median PFS was 6.77 months in the LX group compared with 5.6 months in the TBP group (hazard ratio 0.7955; 95% CI, 0.6632 to 0.9542; log-rank *P* = 0.014; Fig. 1a). In the primary resistant patients, the median PFS was significantly increased from 4.3 months for TBP to 6.8 months for LX (*P* < 0.001; Fig. 1b). In the secondary resistant patients, no significant difference was observed (median PFS: 6.6 months for LX vs 6.3 months for TBP, *P* = 0.8827; Fig. 1c). The best overall response to treatment was not evaluable in 64 patients. We observed no significant difference in the ORR or CBR between the two groups (*P* = 0.822; *P* = 0.224; eTable 1 in Supplement 1).

### First-line treatment

**Fig. 2:**
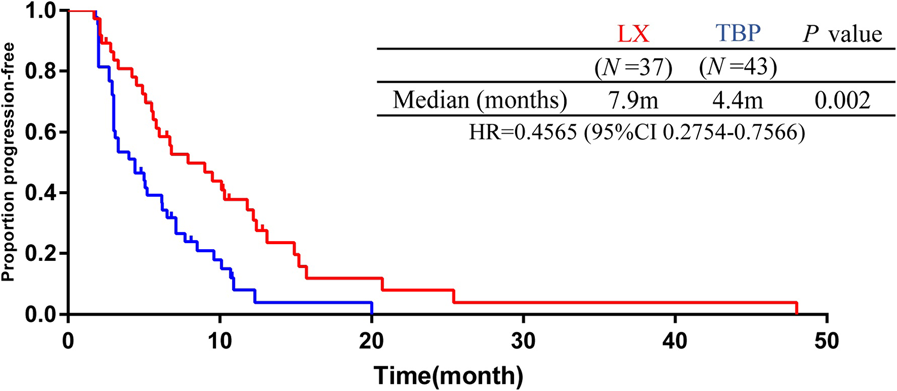
Kaplan-Meier analysis of progression-free survival in first line treatment population

In the TBP group, 3 (7%) patients progressed on (neo) adjuvant trastuzumab therapy and 40 (93%) patients progressed within 12 months after completing (neo) adjuvant therapy. In the LX group, 3 (8.2%) patients relapsed on and 34 (91.8%) patients relapsed within 12 months after the end of (neo) adjuvant trastuzumab treatment. Hence, they are all primary resistant to trastuzumab. The median PFS was 7.9 months in the LX group compared with 4.4 months in the TBP group (hazard ratio 0.4565; 95% CI, 0.2754 to 0.7566; log-rank *P* = 0.002; Fig. 2). A total of 15 patients were not evaluable for best response to treatment. The ORR was significantly increased from 8.3% for TBP to 27.6% for LX (*P* = 0.04). The CBR was significantly improved as well (36.1 to 69%, *P* = 0.008; eTable 2 in Supplement 1).

### Second- and third-line treatment

**Fig. 3:**
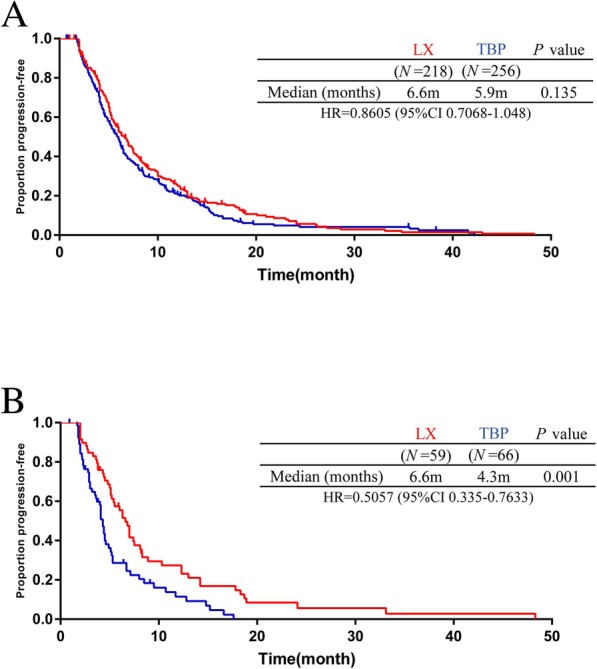
Kaplan-Meier analysis of progression-free survival in second and third line treatment population (**a**) PFS in all patients (**b**) PFS in the primary resistant population

After developing resistance to the trastuzumab-containing treatment, 218 patients received LX, and 256 patients continued using trastuzumab in the later lines. The median PFS was 6.6 months for the LX group compared with 5.9 months for the TBP group (hazard ratio 0.8605; 95% CI, 0.7068 to 1.048; log-rank *P* = 0.135; Fig. 3a). No improvement in median PFS was observed. Median PFS in the primary resistant population increased from 4.3 months for TBP to 6.6 months for the LX group (hazard ratio 0.5057; 95% CI, 0.335 to 0.7633; log-rank *P* = 0.001; Fig. 3b). The best response to treatment was missing in 22 patients in the second-line setting. The differences in the ORR and CBR between the two groups had no significant difference (eTable 3 in Supplement 1). In the third-line setting, 27 patients were not evaluable for best response to treatment. We found no significant difference in ORR or CBR (eTable 4 in Supplement 1).

### Multivariate analysis

**Fig. 4:**
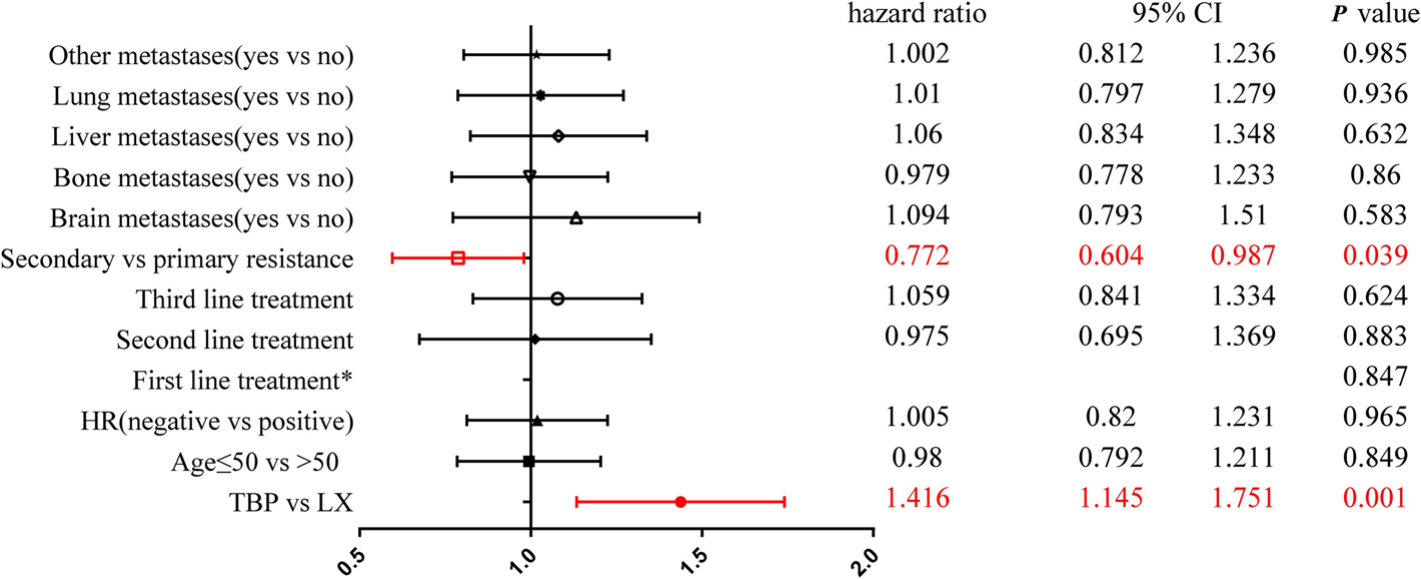
Multivariate analysis for progression-free survival Derived from the Cox regression model. HR hormone receptors status; *Reference group

We carried out a multivariate analysis to investigate whether the anti-HER2 therapy effect was different according to baseline characters. The model included treatment after resistance to trastuzumab, age, hormone receptor status, metastatic sites, and treatment line. We noted that secondary or primary resistance had a differential prognostic effect in trastuzumab treated patients, and the HR for PFS favored patients who were secondary resistant (Fig. 4).

### Central nervous system metastases

Response in the CNS was evaluable in 451 patients. A total of 58 patients had baseline central nervous system metastases. All had received prior local therapy and their details are presented in Table 3. Three patients in the LX group and 4 patients in the TBP group had more than 3 metastatic sites in their brains. In the patients with baseline CNS metastases, we observed 6 cases of progressive disease in the LX group, while in the TBP group, 20 patients progressed. Among the patients without baseline CNS metastases, 2.96% (6/203) and 4.44% (11/248) developed new CNS metastases in the LX and TBP groups, respectively, during the treatment. The CNS progression rates were 5.9 and 12.5%, respectively (*P* = 0.018; Table 4).

**Table 3:** Patients with CNS metastases

	LX	TBP
Patients	(*N* = 24)	(*N* = 34)
Number of brain metastatic sites		
< 3	21(87.5%)	30(88.2%)
≥ 3	3(12.5%)	4(11.8%)
Local treatment		
Radiotherapy (WBRT and/or SRS)	19(79.2%)	28(82.4%)
Neurosurgery with WBRT and/or SRS	5(20.8%)	6(17.6%)

**Table 4:** Central nervous system metastases progression rate

	TBP	LX	*P*
	(*N* = 248)	(*N* = 203)	
CNS as new sites of progression	11	6	
Progression of CNS metastases at baseline	20	6	
CNS progression rate	12.5%	5.9%	0.018

### Safety

The most common adverse events were neutropenia, thrombocytopenia and hand-foot syndrome. A total of 42 (17.8%) patients in the LX group and 61 (20.6%) patients in the TBP group experienced grade 3 or 4 toxicities (*P* = 0.415). The most frequent grade III–IV AEs were diarrhea (5.1%) and hand-foot syndrome (10.2%) in the LX group, while increases of ALT/AST (9.1%) and neutropenia (6.4%) occurred in the TBP group. Treatment-related LVEF decline was observed in 2 patients in the trastuzumab group but was moderate in severity (Table 5). This study was retrospective by nature, and therefore, adverse events may be underestimated.

**Table 5:** Treatment-related adverse events

	LX		TBP	
	(*N* = 236)		(*N* = 296)	
	grade1–2	grade3–4	grade1–2	grade3–4
Neutropenia	24(10.2%)	5(2.1%)	87(29.4%)	19(6.4%)
Febrile neutropenia	4(1.7%)	0(0.0%)	20(6.8%)	4(1.4%)
Thrombocytopenia	12(5.1%)	1(0.4%)	25(8.4%)	3(1%)
Anemia	4(1.7%)	0(0.0%)	40(13.5%)	0(0.0%)
Nausea/Vomiting	60(25.4%)	0(0.0%)	56(18.9%)	8(2.7%)
Diarrhea	92(39.0%)	12(5.1%)	15(5.1%)	0(0.0%)
Cardiac toxicity	0(0.0%)	0(0.0%)	2(0.7%)	0(0.0%)
Rash or erythema	45(19.1%)	0(0.0%)	13(4.4%)	0(0.0%)
ALT/AST increased	28(11.9%)	0(0.0%)	32(10.8%)	27(9.1%)
Hand–foot syndrome	56(23.7%)	24(10.2%)	7(2.4%)	0(0.0%)

Abbreviations: *NCI CTCAE* National Cancer Institute Common Terminology Criteria of Adverse Events

## Discussion

Our study provides evidence that if patients are resistant to trastuzumab, switching to the combination of lapatinib and capecitabine resulted in a longer PFS than continuing the use of trastuzumab. Findings from our analyses suggest that the effect of lapatinib on PFS may be explained by its excellent effect in primary resistant patients.

The results of the current study are in accordance with two small randomized trials comparing capecitabine plus lapatinib with trastuzumab plus lapatinib as treatment for patients progressing on trastuzumab-containing therapy. An analysis of 86 women who were HER-2 positive, had locally advanced breast cancer or metastatic breast cancer (MBC), and developed resistance to trastuzumab, demonstrated that the trastuzumab combined with capecitabine led to a not significantly inferior PFS compared with lapatinib, with a median PFS (7.1 months on LX vs 6.1 months on HX, HR 0.81, 90% CI 0.55–1.21, *P* = 0.39 )[24]. These data are supported by study results from Bian et al., .who randomly assigned 120 HER-2 positive MBC patients with resistance to trastuzumab in a 1:1 ratio to receive capecitabine with either trastuzumab or lapatinib, and reported a median PFS (4.5 months vs 6 months, HR = 0.61, 95% CI: 0.42–0.88, *P* = 0.006 )[25]. They found that 30% of patients in the trastuzumab group and 55% in the lapatinib group experienced a PFS longer than 6 months. Consistent with those reports, our study suggests that patients can respond to further HER2-directed regimens after the development of resistance to HER2-directed therapy. The optimal anti-HER2 treatment for patients who do not respond to trastuzumab treatment in clinical practice is lapatinib when pertuzumab /T-DM1 is not available.

Our findings differ in part from two studies that compared tyrosine kinase inhibitors with trastuzumab for treating HER2-overexpressing metastatic breast cancer. In the LUX-Breast 1 tria l[26], an oral irreversible ErbB family blocker, afatinib, combined with vinorelbine, resulted in a similar PFS as trastuzumab plus vinorelbine in women with HER2-positive metastatic breast cancer who had progressed on trastuzumab. The median PFS was 5.5 months in the afatinib group and 5.6 months in the trastuzumab group (hazard ratio 1.10 95% CI 0.86–1.41; *P* = 0.43). For patients receiving first-line therapy, PFS did not differ significantly among afatinib and trastuzumab-based therapy (hazard ratio 1.102, 95% CI 0.759–1.600; *P* = 0.61). In the MA.31 trial, PFS was shorter for lapatinib plus taxane compared with trastuzumab plus taxane administered as first-line therapy of metastatic breast cancer (9.0 months vs 11.3 months; HR 1.37 [95% CI 1.13–1.65]; *P* = 0.001 )[27]. The trial was terminated early. However, although afatinib is a second-generation, broader inhibitor of the ErbB family of protein s[28], no randomized trials have been conducted to compare the efficacy of afatinib with lapatinib for women who progressed during trastuzumab treatment. Furthermore, a major difference between the MA.31 trial and our study was that in the MA.31 trial, a large proportion of patients were newly diagnosed with advanced breast cancer and were trastuzumab-naïve. This might affect their survival outcomes.

Lapatinib has a different mechanism of inhibition on HER2 and EGFR signaling compared with trastuzumab. Preclinical evidence suggests non-cross-resistance to trastuzumab and lapatinib. PTEN abrogates phosphatidyl inositol-3-kinase (PI3K), which results in inhibition of Akt signaling. Nonexistent or limited expression of PTEN (phosphatase and tensin homologue deleted on chromosome 10) might be a marker of resistance to trastuzuma b[29]. Previous studies have confirmed PTEN expression has no correlation with response to lapatini b[30]. IGF-1R (insulin-like growth factor receptor) is important for cell proliferation and surviva l[31]. It has been reported that overexpression of IGF-1R predicted resistance to trastuzumab in breast cancer cell s[31–33]. IGF-1R belongs to the tyrosine kinase receptor family, and breast cancer cells that express IGF-1R may still be sensitive to lapatini b[34].

We tried to identify subsets of patients who would derive the greatest benefit from further HER2-directed therapy. To this end, we examined whether the prognosis in the primary resistant patients paralleled those that were secondary resistant to HER2-directed therapy. Indeed, in multiple lines, the data showed that the primary resistant patients who received LX tended to have a longer PFS with statistical significance, while the PFS of secondary resistant patients receiving the TBP regimen was similar to that of the patients receiving the LX regimen. p95 HER2 (a truncated version lacking the extracellular domain) prevents trastuzumab binding and is associated with a poor prognosis. Lapatinib inhibits p95HER2 phosphorylation, while trastuzumab doesn’ t[35]. That may explain why switching to lapatinib was associated with an extended PFS in the primary resistant group.

Unlike primary resistant patients, a clinical benefit has been observed for treatment with trastuzumab-containing regimens among patients with acquired resistance to anti-HER-2 therapy. Trastuzumab might have additional anti-tumor efficacy via an antibody-dependent cellular-cytotoxicity (ADCC) mechanism, by which it induces immune effector cells to kill cancer cell s[36, 37].

We also found patients in the second-line treatment had a higher proportion of trastuzumab beyond progression therapy than those in the third-line setting. The predominant HER-2 targeted therapy in the second-line setting was trastuzumab instead of lapatinib. A plausible reason for these disparities concerns the assumption that the patients were refractory to a prior chemotherapy agent but not to trastuzumab itself. Second, anti-HER2 therapy is expensive and time-consuming, and varying medical insurance policies may contribute to the continued use of trastuzumab.

Breast cancer patients with HER2 overexpression have a greater risk for developing brain metastases, and trastuzumab treatment has emerged as a factor contributing to this ris k[38]. Previous studies have supported the hypothesis that the brain is a ‘sanctuary’ site for the development of metastases due to the limited ability of trastuzumab to penetrate the blood-brain barrier (BBB )[39]. Lapatinib is a small dual tyrosine-kinase inhibitor of HER1 and HER2 with a hypothetical ability to cross the BB B[40]. The combination of lapatinib with capecitabine has central nervous system (CNS) activity for the treatment of patients with HER2-positive brain metastatic breast cancer. Clinical evidence indicates that patients with HER2-positive brain metastases achieve a significant clinical benefit from lapatinib and capecitabine both as single agents and as a combinatio n[41–43]. In the present study, the percentage of patients with central nervous system progression was higher in the TBP group. In addition, the comparison of the CNS progression rates indicates that lapatinib is more effective against brain metastases than trastuzumab. These findings are consistent with the results of a randomized clinical trial that evaluated the effect of neratinib compared with trastuzumab in previously untreated metastatic ERBB2-positive breast cancer. Neratinib, another oral irreversible ERBB family blocker, was associated with fewer central nervous system recurrences (relative risk, 0.48; 95% CI, 0.29–0.79; *P* = 0 .002) and delayed the time to CNS relapses compared with trastuzumab (HR, 0.45; 95% CI, 0.26–0.78; *P* = 0.004 )[44]. In the EMILIA trial, there was modest activity of lapatinib plus capecitabine against CNS recurrences, where 2.0% (9/450) in the T-DM1 group and 0.7% (3/446) in the LX group developed new brain metastase s[22, 45]. It appears that switching patients with brain metastases to lapatinib-containing treatment regimens more effectively prevents brain lesion progression.

It should be noted that there were a few limitations to our study. First, it is a retrospective study, and there may be potential imbalances in factors contributing to patient prognosis and patient heterogeneity in terms of treatment. For example, women who switched to lapatinib were younger and more likely to achieve antitumor activity with the new anti-HER2 regimen. Second, the inclusion of patients who received chemotherapy and trastuzumab sequentially or concomitantly may affect the outcomes. Third, some data could not be extracted from the medical records or were missing.

## Conclusions

In conclusion, these data confirm that after developing resistance to trastuzumab, patients can still derive benefit from HER-2 targeted therapy. The combination of lapatinib and capecitabine results in prolonged survival compared with TBP in patients with prior trastuzumab exposure.

## Supplementary information

**Additional file 1.**

## Data Availability

The datasets and the analyses of the current study are available from the corresponding author on reasonable request.

## Abbreviations

ADCC: Antibody-dependent cell-mediated cytotoxicity
CI: Confidence interval
CR: Complete response
CTCAE: Common Terminology Criteria for Adverse Events
DCR: Disease control rate
ECOG: Eastern Cooperative Oncology Group
EGFR: Epidermal growth factor receptor
FDA: Food and Drug Administration
HER2: Human epidermal growth factor receptor 2
HR: Hazard ratio
IGF-1R: Insulin-like growth factor-1 receptor
LC or LX: Lapatinib plus capecitabine
mTOR: Mammalian target of rapamycin
NCCN: National Comprehensive Cancer Network
NCI CTC: National Cancer Institute Common Terminology Criteria
ORR: Objective response rate
OS: Overall survival
PD: Progressive disease
PFS: Progression-free survival
PI3K: Phosphatidyl inositol 3-kinase
PR: Partial response
RECIST: Response Evaluation Criteria in Solid Tumors
SD: Stable disease
SRS: Stereotactic radiosurgery
TBP: Trastuzumab beyond progression
WBRT: Whole brain radiotherapy
