# Supplementary material for: Lapatinib in combination with capecitabine versus continued use of trastuzumab in breast cancer patients with trastuzumab-resistance: a retrospective study of a Chinese population
Source: BMC Cancer. 2020 Mar 29;20:255. doi: 10.1186/s12885-020-6639-4 (PMC7104485; doi:10.1186/s12885-020-6639-4)
Supplement: Supplementary file 1 — Additional file 1. [file 12885_2020_6639_MOESM1_ESM.doc]

| eTable1 | | | |
| --- | --- | --- | --- |
|  | LX | TBP | *P* |
|  | *N*=228 | *N*=262 |  |
| CR | 5(2.2%) | 2(0.8%) |  |
| PR | 43(18.9%) | 51(19.5%) |  |
| SD≥6m | 88(38.6%) | 89(34%) |  |
| ＜6m | 66(28.9%) | 78(29.8%) |  |
| PD | 26(11.4%) | 42(16%) |  |
| ORR | 48(21.1%) | 53(20.2%) | 0.822 |
| CBR | 136(59.6%) | 142(54.2%) | 0.224 |
| eTable2 |  |  |  |
|  | LX | TBP | *P* |
|  | *N*=29 | *N*=36 |  |
| CR | 1(3.4%) | 0 |  |
| PR | 7(24.1%) | 3(8.3%) |  |
| SD≥6m | 12(41.4%) | 10(27.8%) |  |
| ＜6m | 8(27.6%) | 15(41.7%) |  |
| PD | 1(3.4%) | 8(22.2%) |  |
| ORR | 8(27.6%) | 3(8.3%) | 0.04 |
| CBR | 20(69%) | 13(36.1%) | 0.008 |
| eTable3 |  |  |  |
|  | LX | TBP | *P* |
|  | *N*=82 | *N*=154 |  |
| CR | 2(2.4%) | 1(0.6%) |  |
| PR | 17(20.7%) | 37(24%) |  |
| SD≥6m | 32(39%) | 54(35.1%) |  |
| ＜6m | 22(26.8%) | 44(28.6%) |  |
| PD | 9(11%) | 18(11.7%) |  |
| ORR | 19(23.2%) | 38(24.7%) | 0.797 |
| CBR | 51(62.2%) | 92(59.7%) | 0.713 |
| eTable4 |  |  |  |
|  | LX | TBP | *P* |
|  | *N*=117 | *N*=72 |  |
| CR | 2(1.7%) | 1(1.4%) |  |
| PR | 19(16.2%) | 11(15.3%) |  |
| SD≥6m | 44(37.6%) | 25(34.7%) |  |
| ＜6m | 36(30.8%) | 19(26.4%) |  |
| PD | 16(13.7%) | 16(22.2%) |  |
| ORR | 21(17.9%) | 12(16.7%) | 0.822 |
| CBR | 65(55.6%) | 37(51.4%) | 0.577 |
